# Multi-dimensional sensorimotor grounding of concrete and abstract categories

**DOI:** 10.1098/rstb.2021.0366

**Published:** 2023-02-13

**Authors:** Briony Banks, Louise Connell

**Affiliations:** ^1^ Department of Psychology, Lancaster University, Fylde College, Bailrigg, Lancaster LA1 4YF, UK; ^2^ Department of Psychology, Maynooth University, Maynooth, Co. Kildare, Ireland

**Keywords:** abstract concepts, semantic categories, sensorimotor grounding, category production

## Abstract

Semantic categories, and the concepts belonging to them, have commonly been defined by their relative concreteness, that is, their reliance on perception. However, sensorimotor grounding must be regarded as going beyond the basic five senses and incorporate a multi-dimensional variety of perceptual and action experience. We present a series of exploratory analyses examining the sensorimotor grounding of participant-produced member concepts for 117 categories, spanning concrete (e.g. *animal* and *furniture*) and highly abstract (e.g. *unit of time* and *science*) categories. We found that both concrete and abstract categories are strongly grounded in multi-dimensional sensorimotor experience. Both domains were dominated by vision and, to a lesser extent, head movements, but concrete categories were more grounded in touch and hand–arm action, while abstract categories were more grounded in hearing and interoception. Importantly, this pattern of grounding was not uniform, and subdomains of concrete (e.g. ingestibles, animates, natural categories and artefacts) and abstract (e.g. internal, social and non-social) categories were grounded in different profiles of sensorimotor experience. Overall, these findings suggest that the distinction between abstract and concrete categories is not as clearcut as ontological assumptions might suggest, and that the strength and diversity of sensorimotor grounding in abstract categories must not be underestimated.

This article is part of the theme issue ‘Concepts in interaction: social engagement and inner experiences’.

## 1. Introduction

Categorization is a fundamental part of human cognition, allowing us to group together distinct concepts and treat them as equivalent. However, most work on categorization has concentrated on concrete categories (e.g. *animals*, *fruit* or *tools*), and our understanding of abstract categories (e.g. *science, emotions* or *social relationships*) is lacking in comparison. In particular, little is known about the specifics of how abstract categories are grounded in perceptual and action experience, and how their grounding compares with that of concrete categories. Our use of the term ‘grounding’ in the present paper relates to the classic sense of the symbol grounding problem [1,2] and its directional counterpart the transduction problem [3,4], which refers to the way in which symbolic tokens such as words cannot derive their meaning (i.e. their semantics cannot be learned and represented) solely via association with one another in a closed system. Rather, they must outsource their meaning to a more direct, non-symbolic format that relates to experience with the word's referent. We concentrate here on sensorimotor grounding; that is, how perception and action systems provide grounding for words.

Although a range of views exist regarding the centrality of grounding in cognition, from unembodied (no grounding) to strong embodiment (completely grounded; see [5]), many recent theories concur that concepts are represented through both sensorimotor and linguistic information (e.g. [6–9]). These theories hold that, in addition to language, our sensory and motor experiences contribute to the mental representation of concepts, via multiple sensory modalities (e.g. vision [10], touch [11], hearing [12], smell [13] and interoception [14]) and actions with different parts of the body (e.g. the face, arms and legs [15,16]). Accessing conceptual representations involves partial simulations of these perceptual and motor experiences: that is, some of the perceptual and motor systems involved in experiencing a concept are reactivated when, for example, reading the concept's label. Evidence for such theories comes from a wide range of sources, including behavioural [11,17–20], neuroimaging [15,16,21,22] and neuropsychological studies [12,23,24], which have demonstrated that simulations are automatic and unconscious [25,26] and can contribute to our processing and understanding of concepts [27,28]. Collectively, this evidence supports a role for sensorimotor systems in conceptual representation and processing.

Current theories of abstract concepts, in particular, also acknowledge a role for sensorimotor experience along with language (e.g. [29,30]), emotions and internal experiences (e.g. [30,31]), social interactions (e.g. [32]) and situations and events (e.g. [33]). Many theories assume that abstract concepts are more weakly grounded than concrete concepts [9,33,34], and that they are, for example, predominantly or solely represented through linguistic associations (e.g. [9,35,36]). Empirical work has shown, however, that abstract concepts are at least sometimes strongly grounded in perceptual and action experience. For example, the perceptual strength of a concept's dominant modality predicts lexical processing for abstract as well as concrete concepts ([37], see also [38]), while abstract words that evoke a richer sensory experience produce faster response times in semantic categorization [39]. Indeed, Banks *et al*. [40] found that sensorimotor information (composited across multiple dimensions) was equally predictive of responses in abstract category production (e.g. name as many members of the category *science* as possible) as in concrete category production (e.g. name as many *animals* as possible).

Particular types of sensorimotor experience have also been associated with certain subdomains of abstract concepts. Interoceptive experience (i.e. sensations inside the body) is more strongly rated for abstract concepts than concrete and dominates emotion concepts [14]. Facial muscles are activated when making semantic decisions about emotion words [41], and the motor cortex is somatotopically recruited in emotion word processing in the same way as for action words [42]. Mouth movements are more important to abstract concepts than concrete [43,44], but are particularly associated with internal states and social concepts [45], while hand movements are particularly important to numerical and mathematical concepts [46–49]. Collectively, these findings suggest that at least some abstract concepts may be as grounded in sensorimotor experience as concrete concepts, but that the manner of this grounding (i.e. which perceptual modality or action effector) may depend on the exact type of abstract concept in question.

### (a) The current study

Despite the evidence summarized above, many questions remain regarding the grounding of semantic categories from both conceptual domains. First, are categories of abstract and concrete concepts grounded to the same extent? Second, are concrete and abstract categories equally grounded in all sensorimotor dimensions, or does the type of grounding differ between the two domains? Concrete concepts have often been regarded as strongly grounded in vision and touch, but considering multiple dimensions of perception and action is important to fully understand their representation. Moreover, as noted above, the manner of grounding may differ between subdomains, particularly because the abstract domain is arguably more complex and heterogeneous than concrete (e.g. [47,50]). Third, is sensorimotor grounding equally important to the cohesion of both abstract and concrete categories? Previous work has suggested that the members of abstract categories have less perceptual information in common than do the members of concrete categories [51], and hence do not rely on shared sensorimotor grounding in order to cohere as a category. Examining the grounding of concrete and abstract categories, across multiple sensory and motor dimensions, may provide insights into these questions.

In a series of exploratory studies, we therefore examined the multi-dimensional sensorimotor grounding of concrete and abstract categories generated from a large set of category production norms. Studying semantic categories via tasks such as category production (a.k.a. verbal fluency) provides a valuable source of information regarding the organization and representation of conceptual knowledge and—because category members are freely produced by participants—does so with a higher degree of ecological validity than when researchers pre-select category members for analysis. While the majority of such research has focused on concrete categories (e.g. [52]), recent category production norms by Banks & Connell [53] cover a large number of categories in both abstract and concrete domains and thus form the basis of our present analyses. Operationalizing multi-dimensional sensorimotor grounding, particularly for abstract concepts, is potentially complex. While some research on grounding has used concept feature lists (e.g. [33]), we opted not to do so because it restricts data to aspects of representation that can be easily verbalized, not all of which have transparent sensorimotor grounding. Instead, we employed the Lancaster Sensorimotor Norms [54], which provide continuous ratings of experiential strength across 11 individual sensorimotor dimensions, where each dimension corresponds to a discrete area of sensory or motor cortical processing. In this context, sensorimotor strength ratings [54] reflect the degree to which a given referent concept can be perceived through each perceptual modality, or can be experienced by performing an action with each effector. As such, a multi-dimensional profile of sensorimotor strength approximates the distributed neural representation of a concept across the sensory and motor cortices, and hence approximates *how* the perception and action systems provide distributed grounding for words.

Our first aim was to compare the overall grounding of concrete and abstract categories (i.e. are they grounded in sensorimotor experience to the same extent? Study 1). Second, we compared the specific types of perceptual and action experience that are present across the two domains, and particularly for subdomains of both concrete and abstract categories (Study 2). Finally, we examined the diffuseness of concrete and abstract categories (i.e. within individual categories, how much do member concepts share sensorimotor information? Study 3). To this end, we analysed sets of member concepts spontaneously produced by participants for 117 concrete and abstract categories [53], using ratings of sensorimotor experience across 11 dimensions [54] to examine the multi-dimensional nature of concept grounding. As all analyses were exploratory, we report descriptive statistics only. All datasets and code for studies 1–3 are openly available [55].

## 2. Study 1. Are abstract categories as strongly grounded as concrete categories?

In our first study, we investigated whether concrete and abstract categories were grounded in sensorimotor experience to a similar extent, by examining how strongly their member concepts were experienced via perception and action.

### (a) Method

#### (i) Material

We analysed a set of category production norms [53] that comprised 67 concrete and 50 abstract categories, where each category had been classified as abstract versus concrete based on previous literature and/or WordNet classifications of the category labels.^1^ The 117 categories covered a range of taxonomic levels and category types (e.g. living versus non-living, animate versus inanimate, natural versus artefact), including many categories frequently studied in the categorization literature (e.g. *animal, furniture* and *emotion*) as well as more novel abstract categories (e.g. *personal quality* and *statistical term*), thus providing a large range of categories for comparison. The member concepts of each category in the norms were acquired from a sample of 60 UK-based native speakers of English who completed a computer-based category production (a.k.a. verbal fluency) task. Each participant completed the task for 39 categories, and verbally named as many category members as possible within 60 s per category. Idiosyncratic responses (i.e. category members produced by only one participant) were excluded, and responses with the same core referent were grouped under the most frequently produced label (e.g. for the category *emotion*, both *happy* and *happiness* were grouped as *happy*). The full dataset comprised 2445 category–member items (i.e. each member concept paired with its category label; e.g. *animal–cat*); see [53] for full descriptive statistics. We analysed 2082 items for which sensorimotor ratings were available: 784 abstract category members (e.g. *unit of time–seconds*; *religion–Catholicism*) and 1298 concrete category members (e.g. *furniture–chair*; *vegetable–broccoli*).

To represent sensorimotor grounding, we used Lynott *et al*.'s Lancaster Sensorimotor Norms [54], where each concept was rated according to the extent to which it was experienced via six perceptual modalities (vision, hearing, taste, smell, touch and interoception) and five action effectors (hand–arm, foot–leg, head, mouth and torso). Specifically, we took a composite measure of sensorimotor strength for each item, Minowski-3 distance from the origin, which represented experience in all 11 dimensions but with an attenuated influence of weaker dimensions, and which Lynott *et al*. [54] found to be the best composite measure for predicting word recognition. Composite sensorimotor strength therefore ranged in theory from 0 (not experienced at all in any sensorimotor dimension) to 11.12 (experienced greatly through all 11 sensorimotor dimensions). As Lynott *et al*.'s sensorimotor strength ratings were produced for American English, but our items from the Banks & Connell category production norms were for British English, we matched each item to its equivalent American English term where necessary to extract ratings. For example, we matched equivalent spellings (e.g. item *theatre* → ratings for *theater*) and dialectal terms (e.g. *football* → *soccer*; *wrench* → *spanner*). Plural items were also matched to singular equivalents (e.g. *oranges* → *orange*), abbreviated items were matched to their full version (e.g. *sci-fi* → *science fiction*) and quantifiers were ignored (e.g. *one-eighth* → *eighth*). Polysemous items were matched to their category-specific sense if that term was available in the sensorimotor norms, (e.g. *apple* as a member of *tree* → *apple tree*; *sprouts* as a *green vegetable* → *Brussels sprouts*). All matched terms are listed in the data file [55].

#### (ii) Design and analysis

We explored the relative strength of sensorimotor grounding of abstract and concrete categories in two ways, first at a domain level by comparing sensorimotor strength of all items (i.e. all category members across all abstract versus concrete categories), and then at a category level by calculating mean sensorimotor strength per category and ranking categories from weakest to strongest, observing where in the rank order abstract versus concrete categories occurred.

### (b) Results and discussion

Both abstract and concrete domains were strongly grounded in sensorimotor experience (figure 1), with very similar ranges of composite sensorimotor strength (1.71–8.06 for concrete, 1.55–7.45 for abstract). That is, both abstract and concrete categories had member concepts that were quite weakly (e.g. *nanosecond* as a *unit of time*, *hydrogen* as a *chemical element*) and strongly (e.g. *pain* as a *symptom of illness*, *shower* as a *bathroom fixture*) grounded. On average, however, abstract categories had slightly lower sensorimotor strength (*M* = 4.79, s.d. = 1.0) than concrete categories (*M* = 5.23, s.d. = 0.81), a difference of approximately 4.0% on the composite sensorimotor strength scale.

**Figure 1.**
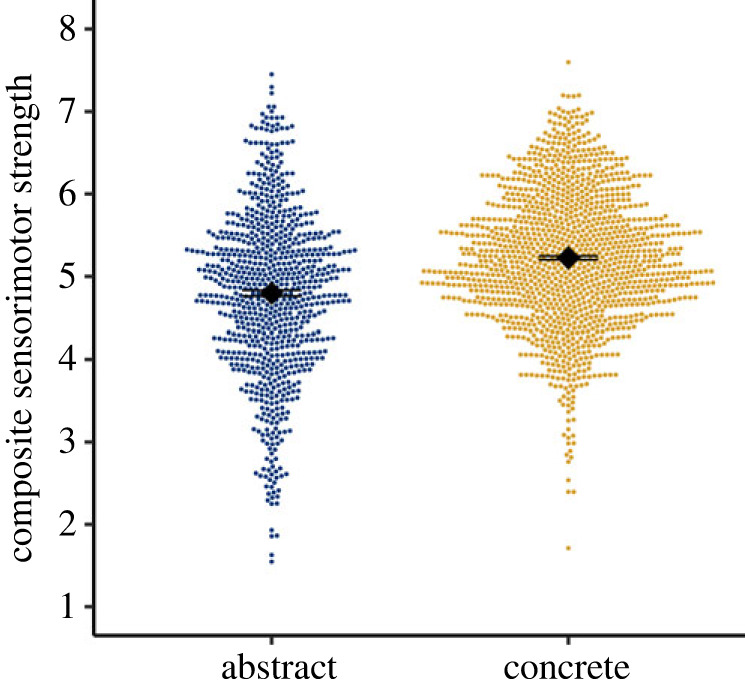
Composite sensorimotor strength of abstract and concrete category members. Data points show individual category members; black diamonds show the mean across all category members per domain; error bars show ± 1s.e. (Online version in colour.)

When examining mean sensorimotor strength per category, the most strongly grounded categories were concrete (e.g. *green vegetable, fruit*; mean rating > 6) and the weakest were abstract (e.g. *day of the week*, *unit of time*; mean rating < 3). Nonetheless, the distinction between concrete and abstract categories was not as clear as might be expected (figure 2). Many abstract categories were ranked highly for their strong sensorimotor grounding (e.g. *racket sport*, *symptom of illness*, *social gathering*, *art form*, *positive emotion*) and were comparable in their mean sensorimotor strength to common concrete categories (e.g. *vegetable, musical instrument*, *tool*, *furniture*, *farm animal*). Similarly, many concrete categories were ranked low for their relatively modest sensorimotor strength (e.g. *chemical element*, *snake*, *metal*, *gemstone* and *religious building*) and were comparable to several clearly abstract categories (e.g. *religion*, *statistical term*, *military title* and *geometric shape*).

**Figure 2.**
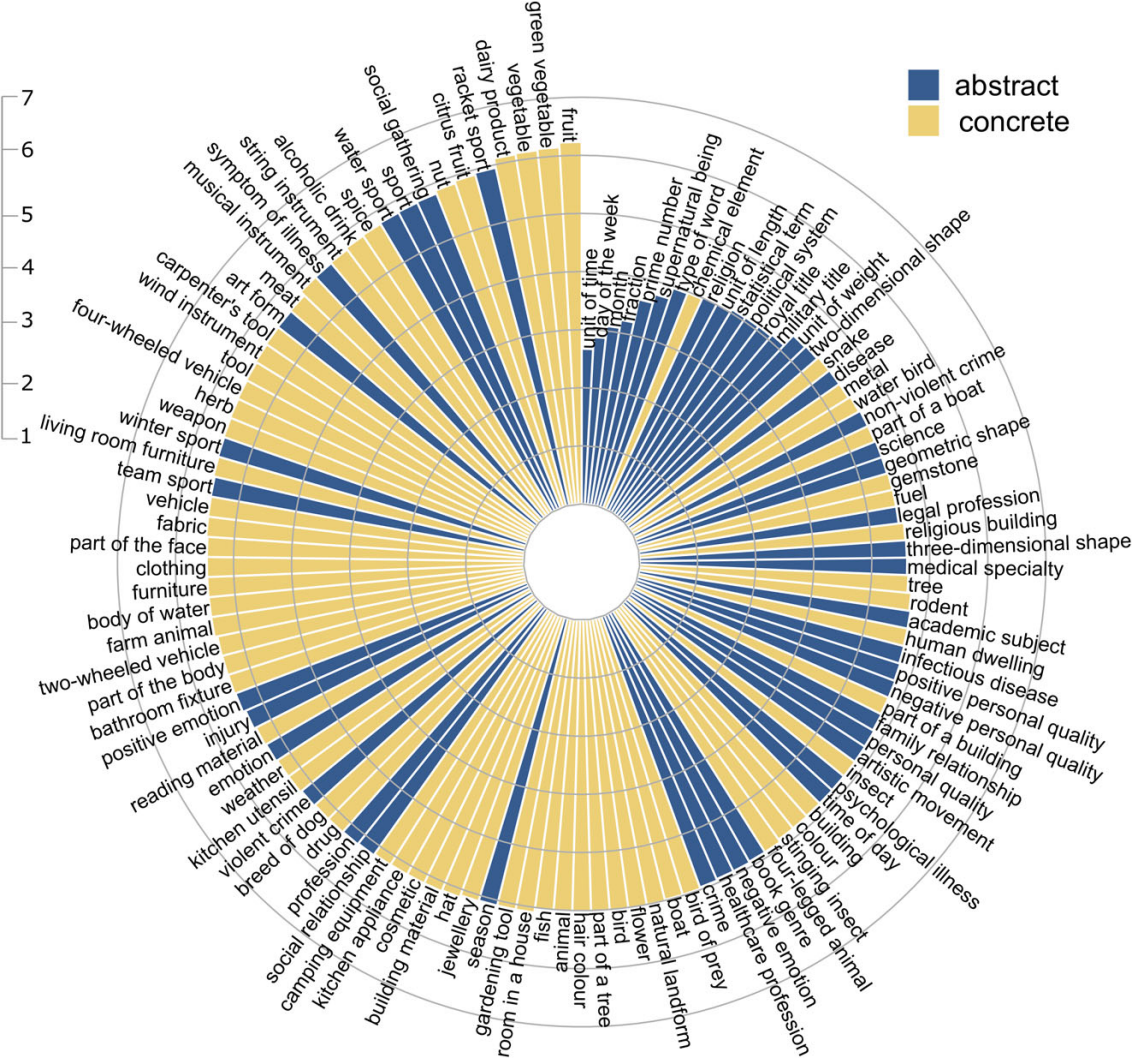
Mean composite sensorimotor strength per category, ordered clockwise from low to high. (Online version in colour.)

These results indicate that both abstract and concrete categories are grounded in sensorimotor experience, albeit to a slightly weaker extent for abstract categories when considered as an overall domain, consistent with some previous findings (e.g. [37]). However, when the strength of sensorimotor grounding is considered for individual categories, the concrete–abstract dichotomy is no longer clearly apparent.

## 3. Study 2. Are abstract and concrete categories grounded via different sensory modalities and action effectors?

Study 1 found that many abstract and concrete categories have equivalent strength of sensorimotor grounding. We next explored whether they differ in *how* they are grounded—that is, whether the overall domains are grounded via different perceptual modalities and/or different action effectors, and whether different subdomains of abstract and concrete categories have distinctive sensorimotor grounding.

### (a) Method

#### (i) Material

All items were the same as in Study 1. Rather than a single composite rating of sensorimotor strength to summarize grounding per item, we took Lynott *et al*.'s [54] ratings in 11 separate dimensions: perceptual experience via six modalities (vision, hearing, taste, smell, touch and interoception) and action experience via five effectors (hand–arm, foot–leg, head, mouth and torso). Sensorimotor strength ratings ranged from 0 (not at all experienced in that dimension) to 5 (experienced greatly in that dimension).

#### (ii) Design and analysis

We first explored broad differences at the domain level, comparing how abstract and concrete categories were grounded in each of the 11 sensorimotor dimensions. Our second exploratory goal was to examine subdomains of concrete and abstract categories, to establish whether different categorical types were differently grounded in perceptual and/or action experience. For concrete categories, we explored several candidate subdomains established in the neuropsychological literature (see [56] for a review) to represent the most common high-level dissociations in semantic deficits (i.e. living/non-living, animate/inanimate, biological/non-biological, natural/artefact and food/non-food) and separately examined others that are frequently ambiguous in their deficit patterns (i.e. musical instruments and parts of the body). We broadened the subdomain of *food* to cover anything that is taken into the body for ingestion (e.g. foodstuffs, drinks and drugs) and relabelled it as ingestible/non-ingestible for clarity. Note that while many of these subdomains largely overlap, they differ in the characterization of certain categories (e.g. *flowers* are living, biological and natural, but inanimate and non-ingestible; *gemstones* are natural but non-living, inanimate, non-biological and non-ingestible). For abstract categories, we explored high-level subdomains that have been proposed in the literature to have different representational structure: internally focused (i.e. relating to inner human experiences and characteristics) versus externally focused categories (i.e. relating to experiences or entities outside the self), and social (i.e. relating to interpersonal interaction) versus non-social categories [45,47,57]. In both cases, we then allocated each semantic category from Banks & Connell [53] to its relevant subdomain (e.g. *furniture* in the inanimate subdomain; *emotion* in the internal subdomain), calculated the mean rating per dimension of all items within each subdomain and examined differences in how each subdomain was grounded compared with its counterpart (e.g. animate versus inanimate; internal versus external). We selected the subdomains that showed the most distinctive differences in sensorimotor grounding and then examined further nesting within each of these subdomains.

### (b) Results and discussion

Abstract and concrete category members share some similarities in how they are grounded (figure 3): both domains are primarily and strongly grounded in visual experience and (moderately) in head movements. Importantly, both domains are grounded to some extent across all 11 dimensions of perceptual and action experience, although to differing degrees. Abstract categories are more strongly grounded than concrete categories in interoception, hearing, and movements of the mouth and head, as well as in—somewhat surprisingly—movements of the torso and foot–leg. By contrast, concrete categories are more strongly grounded than abstract categories in haptic experience and hand–arm movements in particular, as well as in vision, smell and taste. Thus, although multiple dimensions of perceptual and action experience are present in both domains, abstract and concrete categories are overall grounded in different forms of sensorimotor experience.

**Figure 3.**
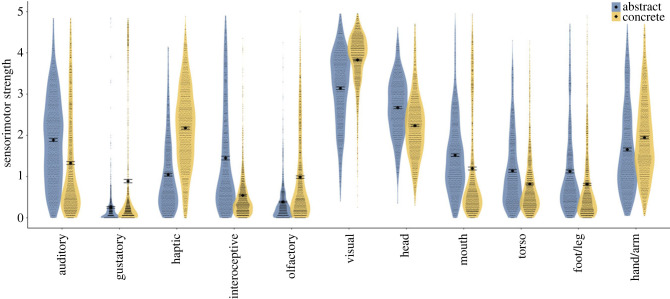
Sensorimotor strength ratings per dimension for abstract and concrete category members. Data points show individual category members; black diamonds show the mean across all category members per dimension; error bars show ± 1s.e. (Online version in colour.)

Figures 4 and 5 present different patterns of sensorimotor grounding for subdomains of abstract and concrete categories, respectively. For abstract categories, all subdomains showed a consistently strong presence of head action, but the clearest distinction was between internally and externally focused subdomains. Internal categories (e.g. *emotion*, *personal quality*, *symptom of illness*, *injury* and *psychological illness*) were most strongly grounded in interoception and head action, and it was the only abstract (or concrete) subdomain not dominated by visual experience. While some individual categories or member concepts in this subdomain were grounded in additional dimensions (e.g. *injury* is grounded in haptic and hand–arm action), the profile of internally focused categories remained evident throughout.

**Figure 4.**
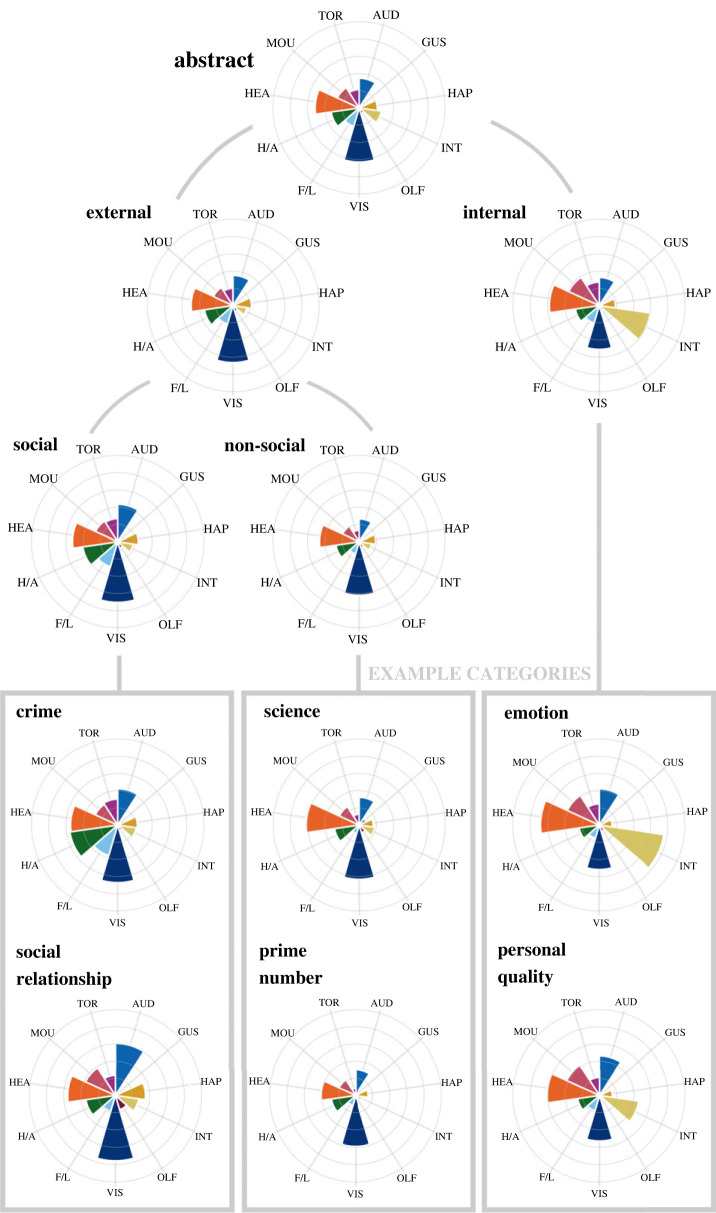
Multi-dimensional profiles of abstract category subdomains, showing mean ratings for 11 dimensions of sensorimotor experience: AUD = auditory; GUS = gustatory; HAP = haptic; INT = interoceptive; OLF = olfactory; VIS = visual; F/L = foot–leg; H/A = hand–arm; HEA = head; MOU = mouth; TOR = torso. (Online version in colour.)

**Figure 5.**
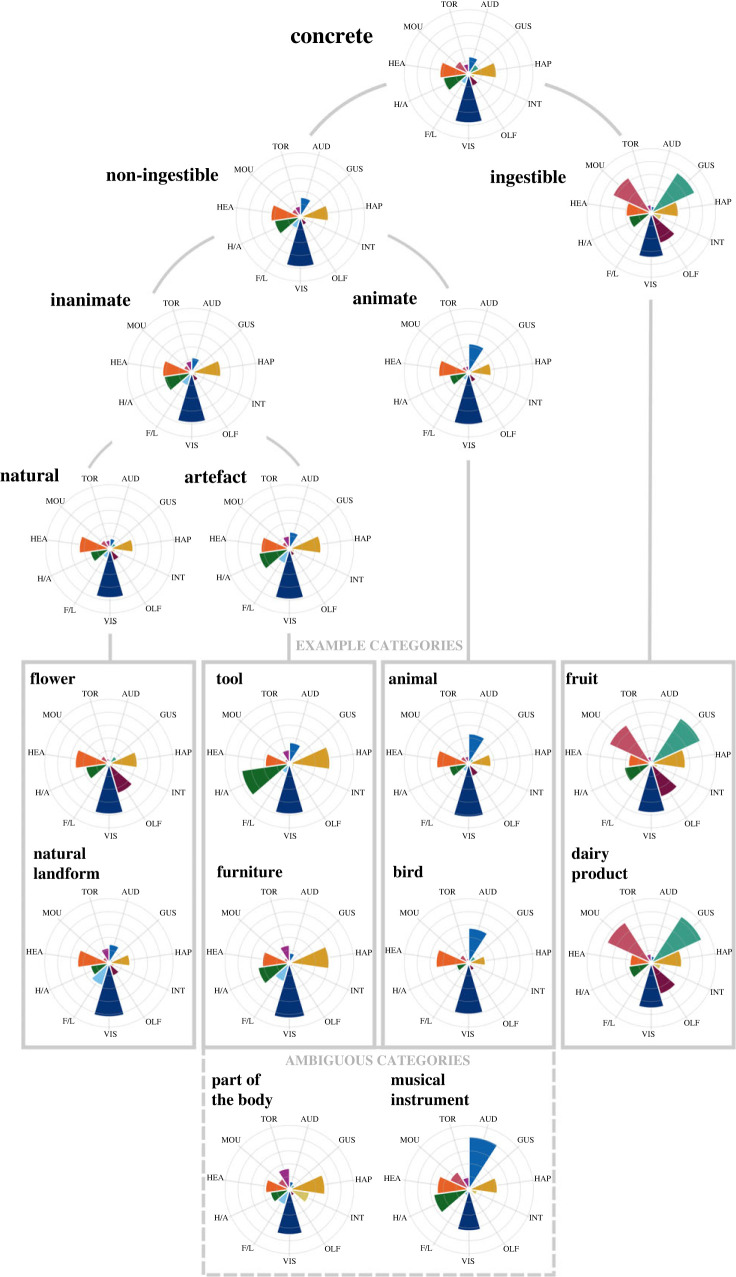
Multi-dimensional profiles of concrete category subdomains, showing mean ratings for 11 dimensions of sensorimotor experience: AUD = auditory; GUS = gustatory; HAP = haptic; INT = interoceptive; OLF = olfactory; VIS = visual; F/L = foot–leg; H/A = hand–arm; HEA = head; MOU = mouth; TOR = torso. (Online version in colour.)

By contrast, external categories were most strongly grounded in vision and head action, with negligible grounding in interoception, and both social and non-social subdomains fitted within this profile. Non-social categories (e.g. *prime number*, *geometric shape*, *science*, *month* and *unit of weight*) followed the same pattern of grounding that exemplifies external categories as a whole, with some individual categories also introducing additional dimensions (e.g. *geometric shape* was also grounded to a limited extent in haptic perception and hand–arm action). Social categories (e.g. *family relationship*, *profession*, *religion*, *sport* and *crime*) generally showed stronger visual and head action grounding than the non-social subdomain, and were also strongly grounded in hearing and to some extent in mouth action. Again, some individual social categories were grounded in additional dimensions, such as *sport*'s use of hand–arm, foot–leg and torso action, which was largely responsible for the presence of these latter dimensions in the abstract domain as a whole.

For concrete categories, all subdomains were dominated by grounding in vision and a consistent, moderate presence of head action. The most striking differences in grounding appeared between things that can be ingested (i.e. all food categories such as *fruit*, *vegetable*, *meat*, *dairy product*, plus *alcoholic drink* and *drug*) and things that cannot, which produced larger distinctions than any other candidate subdomains (see [55]). Ingestible categories were strongly grounded in gustatory, olfactory and mouth–throat action experience (in addition to the general concrete profile of vision and head action), and to some extent on haptic and hand–arm action, but tended to have negligible grounding in auditory or foot–leg action. Two ingestible categories (*alcoholic drink* and *drug*) were additionally grounded in interoception, but otherwise followed the pattern of the subdomain. On the other hand, non-ingestible categories resembled the overall profile of concrete categories in being dominated by vision and head action, with a moderate grounding in haptic and hand–arm action that was stronger than that exhibited by ingestible categories, and a notable absence of grounding in taste or interoception.

Examining non-ingestible categories more closely showed a clear distinction between animate and inanimate subdomains. Animate categories (e.g. *animal*, *bird*, *snake* and *insect*) were dominated by auditory grounding in addition to the ubiquitous visual and head action grounding and had a markedly weaker presence of touch and hand–arm action. By contrast, inanimate categories followed the pattern of the non-ingestible domain and further subdivided into natural/artefact subdomains. Natural categories (e.g. *flower*, *tree*, *body of water* and *weather*) were overall dominated simply by visual and head action grounding, with a weaker presence of touch and hand–arm action than the inanimate subdomain as a whole. Some individual natural categories were also grounded in additional sensorimotor dimensions (e.g. *flower* and *body of water* had some olfactory grounding; *natural landform* had some foot–leg grounding) but never as the dominant means of grounding. Artefact categories (e.g. *clothing*, *furniture*, *tool* and *vehicle*), however, generally followed the overall profile of the inanimate subdomain but with stronger grounding in touch and hand–arm action in addition to vision and head action. Again, while some individual categories or member concepts within the artefact subdomain featured additional grounding in other sensorimotor dimensions (e.g. *vehicle* and *weapon* had some auditory grounding; *vehicle* and *clothing* had some foot–leg grounding), the characteristic profile of the subdomain was generally present throughout.

Lastly, we turned our attention to the ambiguous concrete categories of *parts of the body* and *parts of the face*, which are often assumed to be animate categories but can be impaired or preserved alongside inanimate artefacts in neuropsychological studies (e.g. [58]). In our data, the sensorimotor profiles for *parts of the body/face* more closely resembled the artefact subdomain in their strong haptic but weak auditory grounding. The categories of musical instruments (i.e. *musical instrument*, *wind instrument* and *string instrument*) represent another ambiguous case, in that they are technically artefacts and are sometimes impaired or preserved as such (e.g. [59]), but often instead pattern after animate categories (e.g. [60]). We found that the sensorimotor profiles of musical instruments resembled a hybrid of *both* subdomains, being similar to the animate subdomain in their strong auditory grounding, but also similar to the artefact subdomain in their strong haptic and hand–arm grounding.

Overall, the distinct patterns we observed in these analyses support previous findings that abstract and concrete categorical domains are both strongly grounded in sensorimotor experience [14,37], but—critically—not with a uniform pattern across different subdomains. Moreover, different subdomains of abstract and concrete categories can be characterized by their distinctive profiles of sensorimotor grounding.

## 4. Study 3. Are abstract categories more diffuse than concrete categories?

Studies 1 and 2 showed that abstract and concrete categories differ little in terms of the strength of their grounding, although they are grounded in very different *types* of sensorimotor experience. However, one other important difference may exist between the domains regarding the extent to which sensorimotor grounding contributes to the ability of member concepts to cohere as a category, as previous work has proposed that the members of concrete categories appear to share more overlapping perceptual information than do the members of abstract categories [51]. That is, abstract categories may be more diffuse in their sensorimotor grounding than concrete categories, whereby their member concepts may share relatively little sensorimotor experience in common, and hence are unlikely to rely on grounding for category cohesion. In this final study, we explored the overlap of sensorimotor experience between category members within each individual category in the abstract and concrete domains.

### (a) Method

#### (i) Material

All material was the same as in Study 2, where each category member was represented as an 11-dimensional vector of sensorimotor experience.

#### (ii) Design and analysis

To examine the diffuseness of categories, we used a measure of sensorimotor distance between all the members that composed each category. For example, for the category *animal*, we calculated the distance between *cat* and all other members of that category (e.g. *dog*, *lion*, *rhino,* …) and then did the same for all other category members (e.g. *dog* compared with *lion*, *rhino*, *…*). Specifically, we calculated the sensorimotor distance between category members as the Minkowski-3 distance between their vectors [61], and then calculated the mean inter-member distance for that category. This final measure per category thus reflects the overlap of sensorimotor experience between its member concepts and indicates how ‘diffuse’ the internal structure of each category is in its sensorimotor grounding. Mean sensorimotor distance per category ranged in theory from 0 (i.e. all category members are identical; category is not at all diffuse) to 11.12 (i.e. all category members share no sensorimotor information; category is extremely diffuse).

Our exploratory goal in this study was to examine whether abstract and concrete categories differed in their level of diffuseness by comparing the distribution of mean sensorimotor distance per category in each domain.

### (b) Results and discussion

Most categories were not very diffuse in their grounding, and mean sensorimotor distance between category members differed very little between the two domains (figure 6). Indeed, concrete categories (*M* = 2.25, s.d. = 0.56) were very slightly *more* diffuse than abstract categories (*M* = 2.23, s.d. = 0.61), that is, within concrete categories, member concepts overlapped slightly *less* in sensorimotor experience than did the member concepts of abstract categories. In terms of sensorimotor grounding, the most diffuse categories were found in both domains (e.g. *art form* and *weather*). One particular concrete category, *part of the body*, was an outlier in its diffuse grounding, whereby its member concepts tended to be dominated by different sensorimotor dimensions (figure 7). Many of the least diffuse categories were also found in both domains (e.g. *religion* and *gemstone*), although the absolute lowest-scoring categories were predominantly abstract (e.g. *two-dimensional shape, type of word*). The members of such non-diffuse categories tended to be grounded in the same sensorimotor dimensions and to similar extents, as illustrated in figure 7 for members of the category *religion*.

**Figure 6.**
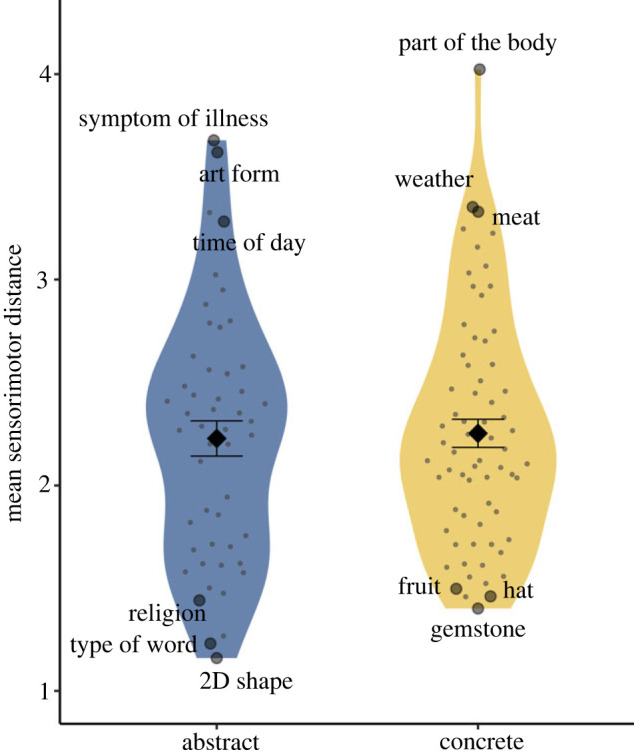
Mean sensorimotor distance between category members for each category, with examples of the most and least diffuse categories labelled by name. Data points show mean individual categories; black diamonds show the mean across all categories per domain; error bars show ± 1s.e. (Online version in colour.)

**Figure 7.**
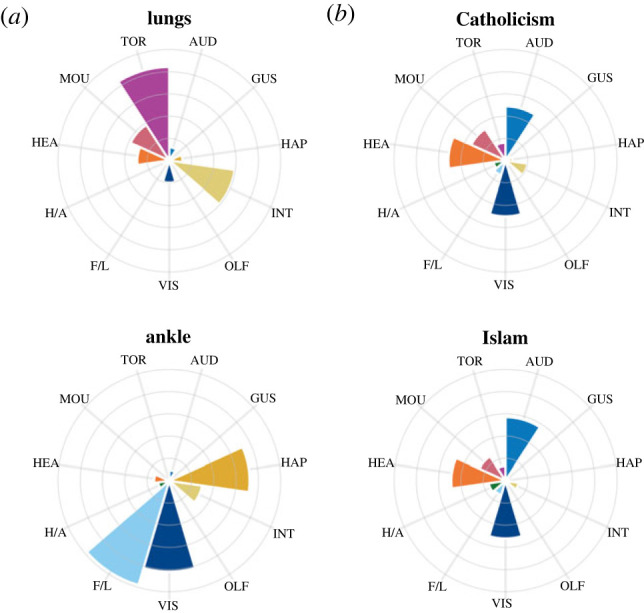
Multi-dimensional profile of sensorimotor strength ratings for sample member concepts from one of the most diffuse categories ((a) *part of the body)* and least diffuse categories ((b) *religion*). (Online version in colour.)

Since a handful of categories were relatively diffuse in their sensorimotor grounding, it begs the question: what makes these categories cohere if their member concepts share little sensorimotor experience in common? Kloos & Sloutsky [51] proposed that such categories cohere on the basis of inclusion rules that are true for members and false for non-members. A similar proposal is that such categories are relational (e.g. [62])—that is, their members are grouped together because they share relational roles or patterns in common rather than because they share intrinsic properties such as sensorimotor experience. Some of the more diffuse categories in our data could indeed be considered to follow a rule or shared relation. For instance, every member of the category *art form* occupies the result role in an act of creation by an *artist* (similarly, every member of *part of the body* occupies a part-of relation with the *body*). However, many non-diffuse categories could also be considered rule-based or relational (e.g. *prime number* and *human dwelling*), so it is not the case that rules or relations replace sensorimotor grounding as a means of category coherence. Nonetheless, it is possible that rules or relational connections, perhaps expressed by language (e.g. [63]), help categories with diffuse sensorimotor grounding to cohere more firmly.

Overall, abstract and concrete categories are about as diffuse as each other in their sensorimotor grounding, that is, categories in each domain are composed of member concepts that generally overlap to the same extent in their strength of grounding in various perceptual modalities and/or action effectors. As a result, sensorimotor grounding is approximately equally important to category coherence in both domains. This pattern is not consistent with Kloos & Sloutsky's [51] suggestion that abstract categories would be *more* diffuse (i.e. their members overlapping less in perceptual information) than concrete categories. However, it should be noted that their proposal was primarily based on counting discrete features in artificial categories and hence may not generalize well to the continuous measures of sensorimotor grounding in real-world semantic categories that we examined here.

## 5. General discussion

In three studies exploring the multi-dimensional sensorimotor grounding of abstract and concrete categories, we found many similarities between the two domains. Categories from both domains were strongly grounded in multiple dimensions of perceptual and action experience, with many categories and their member concepts having equivalent strength of sensorimotor experience. Both concrete and abstract categories were most strongly grounded in vision and had a moderate but consistent grounding in head action. Indeed, abstract and concrete categories were equally diffuse in their grounding, whereby category members overlapped with each other's sensorimotor experience to a similar extent, indicating that sensorimotor grounding was equally important to category coherence in both domains. Overall, we found that abstract and concrete categorical domains were more similar than might be predicted by theories stating that sensorimotor experience is more important to concrete concepts [9,33,34].

The most notable differences we observed were in the relative importance of different sensorimotor dimensions to each domain. Haptic experience and hand–arm movements, and to some extent smell and taste, were far more strongly rated in concrete categories than abstract, while interoception, hearing and to some extent head movements, were more strongly rated in abstract categories. This pattern supports and extends findings from the existing literature regarding the sensorimotor basis of abstract concepts [14,45], but also highlights that concreteness does not necessarily reflect the presence or absence of sensorimotor grounding; rather, it indicates the *type* of grounding typically involved in a domain.

Importantly, our analyses also identified grounding differences in subdomains of both abstract and concrete categories. Supporting theoretical distinctions between subdomains already suggested by the literature (e.g. [45,47,57]), abstract categories fell into subdomains of internally focused (typified by interoceptive and head action grounding) and externally focused (typified by visual and head action grounding, without interoception), the latter of which further split into social (typified by additional auditory grounding) and non-social subdomains. Similarly, concrete categories fell into subdomains of ingestible (typified by strong grounding in taste, smell, vision and mouth action, and to some extent in touch and head action) and non-ingestible (typified by visual, haptic, head action and hand action grounding, and an absence of taste). Non-ingestible in turn fell into further subdomains of animate (typified by additional auditory grounding and weaker haptic and hand–arm grounding) and inanimate, the latter of which subdivided into natural (typified by visual and head action grounding, with relatively weak haptic and hand–arm grounding) and artefact subdomains (typified by touch and hand–arm action in addition to visual and head action grounding). Body parts more closely resembled artefacts, and musical instruments resembled both animate and artefact subdomains. Our findings for concrete categories thus mirrored some of the patterns shown by patients with selective categorical deficits in semantic memory (e.g. [56,58]) purely on the basis of sensorimotor strength (i.e. without functional or other features).

The present findings overall indicate that the concrete–abstract dichotomy is not the optimal way to understand grounding of semantic categories or conceptual representation in general, an argument that has been put forward by several researchers in recent years [14,45,57]. Indeed, defining concepts only in terms of their visual and haptic experience (e.g. as predominantly captured in single-dimension ratings such as imageability [37]) has tended to bias our understanding of the conceptual system and its structure by providing a false impression of a fundamental sensory dichotomy between the domains. We believe researchers should no longer assume the abstract–concrete dichotomy forms a fundamental basis of conceptual structure and should instead attend more closely to exactly what kind of subdomain they are interested in investigating. As we have demonstrated, examining more fine-grained differences between concepts and categories, based on multiple sensorimotor dimensions, can be particularly revealing for how categorical domains are represented. Of course, the importance of sensorimotor grounding does not obviate the rich source of semantic information that can be gleaned from symbolic associations between words (see [64], for review), and linguistic experience is also critical to conceptual representation (e.g. [6–9]). We propose that future work should take into account how multi-dimensionally rich and diverse sensorimotor grounding can be, particularly within the abstract domain, when investigating how language and sensorimotor information interact to inform conceptual structure and processing. Far from being detached from the world of perception and action, many abstract categories relate just as richly to our sensory and motor experiences as do concrete categories.

## Data Availability

All datasets and code are available from the Open Science Framework: https://doi.org/10.17605/OSF.IO/V6SGR [65].

## Endnote

1 As reported in [53], the mean concreteness rating of concrete categories (i.e. concreteness averaged over member concepts) was much higher than the mean concreteness rating of abstract categories.
